# Atherogenic index of plasma and the clinical outcome of patients with acute coronary syndrome: a meta-analysis

**DOI:** 10.1080/07853890.2024.2442532

**Published:** 2024-12-27

**Authors:** Sihai Jiang, Suying Liu, Guie Xiao, Kexuan Liu, Jialin Li

**Affiliations:** a Department of Emergency, Shaoyang Central Hospital, Shaoyang, China; b Department of Cardiovascular Medicine, The Second Affiliated Hospital of Shaoyang University, Shaoyang, China

**Keywords:** Acute coronary syndrome, atherogenic index of plasma, prognosis, major adverse cardiovascular events, meta-analysis

## Abstract

**Background:**

The atherogenic index of plasma (AIP) has been related to an increased risk of coronary artery disease. However, previous studies evaluating the prognostic role of AIP for acute coronary syndrome (ACS) showed inconsistent results. This meta-analysis was conducted to systematically evaluate the association between AIP and the risk of major cardiovascular adverse events (MACE) of patients with ACS.

**Materials and methods:**

Relevant cohort studies were retrieved by searching electronic databases, including PubMed, Web of Science, and Embase. A random-effects model was used to combine the data by incorporating the influence of between-study heterogeneity.

**Results:**

Thirteen datasets from nine cohort studies, involving 10,861 patients with ACS were included in the meta-analysis. Of them, 1546 (14.2%) developed MACE during follow-up. Pooled results suggested that a high AIP at admission was associated with an increased risk of MACE during follow-up (risk ratio [RR]: 1.54, 95% confidence interval [CI]: 1.30–1.82, *p* < 0.001; *I*^2^ = 48%). Subgroup analyses suggested a stronger association between a high AIP and an increased risk of MACE in older patients (mean age ≥60 years, RR: 2.26, 95% CI: 1.78–2.87, *p* < 0.001; *I*^2^ = 0%) than the younger ones (mean age <60 years, RR: 1.30, 95% CI: 1.17–1.44, *p* < 0.001; *I*^2^ = 0%; *p* for subgroup difference <0.001), which fully explained the heterogeneity.

**Conclusion:**

A high AIP is associated with an increased risk of MACE in patients with ACS, particularly for older patients.

## KEY MESSAGES

- A meta-analysis was performed to evaluate the association between AIP and MACE in patients with ACS.
- Results showed that a high AIP is associated with an increased risk of MACE.
- The association was stronger in older patients (mean age ≥60 years) than that in younger patients (mean age <60 years).

## Introduction

Acute coronary syndrome (ACS) represents a serious form of coronary artery disease (CAD), involving the rupture of vulnerable atherosclerotic plaques in the coronary artery and either complete or partial blockage of blood flow due to subsequent thrombosis [1,2]. Currently, ACS has emerged as a leading cause of morbidity and mortality globally [3]. Despite advancements in revascularization techniques, such as percutaneous coronary intervention (PCI), the prognosis for patients with ACS, especially those at high risk, such as older individuals and those with diabetes, remains unsatisfactory [4,5]. As a result, identifying risk factors for poor prognosis in ACS patients has become an important area of clinical research.

Dyslipidemia, especially elevated levels of total cholesterol (TC) and low-density lipoprotein cholesterol (LDL-C), has been identified as a significant risk factor in the development of atherosclerosis [6]. Lowering LDL-C is now crucial in preventing recurrent coronary adverse events for ACS patients [7]. Recent research emphasizes the significance of the atherogenic index of plasma (AIP), a novel parameter based on serum triglyceride (TG) and high-density lipoprotein cholesterol (HDL-C), in the development of CAD [8,9]. An earlier meta-analysis indicated that higher AIP values are independently linked to CAD among adults [10]. However, previous studies on the prognostic role of AIP for ACS have produced conflicting findings [11–19]. Some studies suggested that a high AIP is associated with an increased risk of major cardiovascular adverse events (MACE) in patients with ACS [12,13,15–19], while other studies did not suggest such an association [11,14]. Given this uncertainty, a meta-analysis was performed to comprehensively assess the association between AIP and the risk of MACE for patients with ACS.

## Materials and methods

The Preferred Reporting Items for Systematic reviews and Meta-Analyses (PRISMA) statement (2020) [20,21] was followed in this study. The Cochrane Handbook [21] for systematic review and meta-analysis was referenced throughout the study.

### Literature analysis

Three major electronic databases, PubMed, Web of Science, and Embase were used for a literature search, following a predefined combined search term including (1) ‘atherogenic index of plasma’ OR ‘atherogenic index’ OR ‘AIP’; combined with (2): ‘acute coronary syndrome’ OR ‘myocardial infarction’ OR ‘angina’ OR ‘coronary artery disease’ OR ‘percutaneous coronary intervention’ OR ‘major adverse cardiovascular events’ OR ‘CAD’ OR ‘STEMI’ OR ‘NSTEMI’ OR ‘ACS’ OR ‘AMI’ OR ‘PCI’. Only studies with human subjects and published in English peer-reviewed journals were included. A second-round check-up for the references of the relevant articles was also conducted. The final database search was achieved on 16 January 2024.

### Inclusion and exclusion criteria

The inclusion criteria followed the PICOS principle.

1. P (patients): Patients with confirmed diagnosis of ACS were included, which include ST-segment elevation myocardial infarction (STEMI) and non-ST-segment elevation ACS (NSTEACS). The latter involves non-ST-segment elevation myocardial infarction (NSTEMI) and unstable angina pectoris (UAP).
2. I (exposure): The AIP was measured after hospital admission according to the formula log (TG/HDL-C), and a high AIP at admission was considered as the exposure. The cutoff for defining a high AIP was consistent with the value which was used in the original studies.
3. C (control): Patients with a low AIP at admission were considered the controls.
4. O (outcome): The outcome of the meta-analysis was the incidence of MACE during follow-up compared between ACS patients with the highest *vs.* the lowest category of AIP at admission. Generally, MACE was defined as cardiac death, non-fatal MI, non-fatal stroke, and unplanned repeat revascularization.

We excluded reviews, meta-analyses, studies with AIP analyzed as continuous only, or studies without outcomes of interest. In cases where there was potential overlap in patient population across multiple studies, only the study with the largest sample size was included in this analysis.

### Data collection and quality assessment

Two separate authors conducted a thorough search of academic literature, performed data collection and analysis, and independently assessed the quality of the studies. Any discrepancies that arose were resolved by involving the corresponding author in discussion for final decision-making. Data on study information, design, patient diagnosis, sample size, age, sex, patient diabetic status, proportions of patients who received PCI, timing of AIP measuring, methods for determining the cutoffs of AIP, follow-up durations, numbers of patients with MACE during follow-up, and variables adjusted in the regression model for studying the association between AIP and MACE were gathered. The assessment of study quality was carried out using the Newcastle-Ottawa Scale (NOS) [22], which involved scoring based on criteria including participant selection process, comparability among groups, and validity of outcomes. This scale utilized a rating system ranging from 1 to 9 stars; higher stars indicated better study quality.

### Statistical methods

An association between AIP and MACE in patients with ACS was presented using RR and corresponding 95% confidence interval (CI), comparing the incidence of MACE between ACS patients with the highest *vs.* the lowest category of AIP at admission. Data of RRs and standard errors were calculated based on the 95% CIs or *p*-values, followed by a logarithmical transformation to ensure stabilized variance and normalized distribution [21]. The heterogeneity among studies was assessed using the Cochrane *Q* test and *I*^2^ statistic [23,24], with *I*^2^ > 50% indicating significant statistical heterogeneity. A random-effects model was used for result aggregation considering the influence of heterogeneity [21]. Sensitivity analysis involving the exclusion of one dataset at a time was conducted to assess the robustness of findings [21]. Additionally, multiple subgroup analyses were performed to evaluate the influences of study characteristics on the results, such as study design, subtype of ACS, mean age, proportions of men, patients with diabetes, and patients who received PCI treatment, mean follow-up duration, and analytic model for the association (univariate or multivariate). Medians of continuous variables were selected as the cutoff values for defining subgroups. Publication bias estimation involved constructing funnel plots, initially evaluated through visual inspection for symmetricity before being analyzed using Egger’s regression test [25], where *p* < 0.05 indicates statistical significance. These analyses were conducted utilizing RevMan Version 5.1 (Cochrane Collaboration, Oxford, UK) and Stata software version 12 (Stata Corporation, College Station, TX, USA).

## Results

### Study inclusion

**Figure 1.**
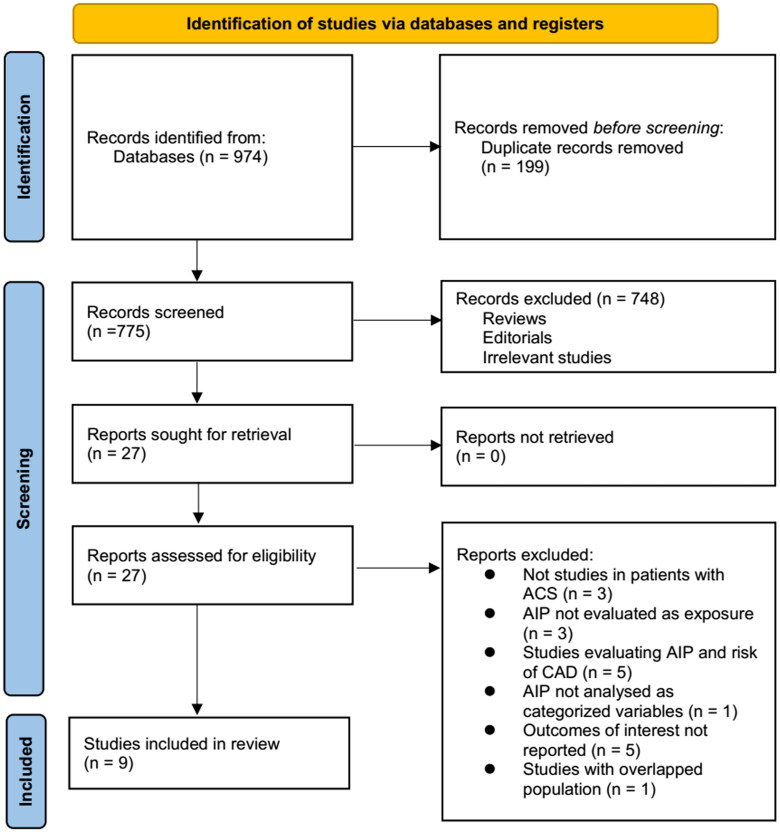
Process for conducting literature search and identifying studies.

The process of selecting relevant studies for inclusion in the meta-analysis is depicted in Figure 1. Initially, 974 potentially pertinent records were identified through thorough searches of three databases. Among these, 199 were removed due to duplication. Subsequent screening based on the titles and abstracts resulted in the exclusion of an additional 748 studies that did not align with the aim of the meta-analysis. The full texts of the remaining 27 studies underwent independent review by two authors, leading to the removal of a further 18 studies for various reasons detailed in Figure 1. Ultimately, nine cohort studies remained [11–19] that were considered suitable for subsequent quantitative analyses.

### Overview of the studies’ characteristics

Table 1 presents the summarized characteristics of the included studies. Overall, four prospective cohort studies [11,13–15] and five retrospective cohort studies [12,16–19] were included in the meta-analysis. These studies were published between 2016 and 2024, and were performed in Indonesia, China, Turkey, and India. All of the studies included adult populations with ACS. The mean ages of the patients were 57.4–61.3 years, and the proportions of men were 66.9–81.9%. PCI was administered for all the included patients in five studies [12–15,18]. The assessment for AIP was achieved within 24 h after admission or before PCI. Methods for defining the cutoff of AIP varied among the included studies, such as using cutoff defined by previous studies [11,19], receiver operating characteristic curve analysis [16], or median [17,18], tertiles [13–15], or quartiles [12] of AIP values. The lengths of follow-up duration varied from within hospitalization to 30.5 months. During the follow-up, 1546 (14.2%) of the included patients developed MACE. The association between AIP and MACE was evaluated with univariate analysis in two studies [11,19], and in multivariate analysis in seven studies [12–18]. Variables, such as age, sex, cardiovascular risk factors, comorbidities, and cardiovascular medications were adjusted to varying degrees among the included studies. The NOS of the included studies were six to nine stars, suggesting overall moderate to good study quality (Table 2).

**Table 1.** Summary of study characteristics.

Study	Country	Study design	Diagnosis	Sample size	Mean age (years)	Male (%)	DM (%)	PCI (%)	Timing of AIP assessment	AIP cutoff determination	Follow-up duration (months)	No. of patients with MACEs	Variables adjusted
Hartopo 2016	Indonesia	PC	STEMI and NSTEMI	277	57.8	81.9	27.8	26.7	Within 24 h of hospital admission	Previous study derived	During hospitalization	66	None
Ma 2020	China	RC	ACS	826	61.3	72.7	100	100	After admission prior to PCI	Q4:Q1	30.5	198	Age, sex, BMI, CV risk factors, cardiac failure, SCr, LDL-C, LVEF, pre-PCI, subtype of ACS, CAD severity, and lesion characteristics
Zheng 2022	China	PC	ACS	3432	57.4	79.4	0	100	After admission prior to PCI	T3:T1	28	186	Age, sex, BMI, classification of ACS, CV risk factors, pre-PCI, pre-CABG, pre-MI, COPD, PAD, CTO, TVD, SCr, albumin, LM involved, and CV medications
Shao 2022	China	PC	ACS	1694	60	76.5	45.8	100	After admission prior to PCI	T3:T1	31	345	Age, sex, BMI, CV risk factors, pre-MI, pre-PCI, CKD, subtype of ACS, GRACE risk score, hs-CRP, SYNTAX score, complete revascularization, and CV medications
Liu 2023	China	RC	UAP	1096	59.5	69.9	0	26.9	After admission	ROC curve analysis derived	26.3	141	Age, sex, BMI, CV risk factors, pre-PCI, TC, LDL-C, eGFR, HbA1c, CRP, and CV medications
Wang 2023	China	RC	ACS	1133	58.6	85.3	42.8	100	The next day of admission	Median	26	88	Age, sex, BMI, CV risk factors, pre-MI, pre-stroke, TC, LDL-C, HbA1c, SUA, and oral hypoglycemic agents
Ozen 2023	Turkey	RC	ACS	558	59	75.8	38.2	40	After admission	Median	12	137	Age, sex, BMI, CV risk factors, SCr, LVEF, CRP, and TVD
Kan 2023	China	PC	ACS	1725	60.2	75	46.1	100	After admission prior to PCI	T3:T1	19.7	357	Age, sex, BMI, CV risk factors, subtype of ACS, CKD, pre-MI, pre-PCI, LDL-C, hs-CRP, SYNTAX score, and complete revascularization
Karre 2024	India	RC	ACS	120	60.6	71.7	31.9	87.5	Within 24 h of hospital admission	Previous study derived	12	28	None

PC: prospective cohort; RC: retrospective cohort; STEMI: ST-segment elevation myocardial infarction; NSTEMI: non ST-segment elevation myocardial infarction; ACS: acute coronary syndrome; UAP: unstable angina pectoris; DM: diabetes mellitus; PCI: percutaneous coronary intervention; AIP: atherogenic index of plasma; Q: quartile; T: tertile; ROC: receiver operating characteristic; MACE: major adverse cardiovascular events; BMI: body mass index; SCr: serum creatinine; CV: cardiovascular; LDL-C: low-density lipoprotein cholesterol; LVEF: left ventricular ejection fraction; pre-PCI: previous percutaneous coronary intervention; CAD: coronary artery disease; CABG: coronary artery bypass graft; MI: myocardial infarction; COPD: chronic obstructive pulmonary disease; PAD: peripheral artery disease; CTO: chronic total occlusion; TVD: three-vessel disease; LM: left main; GRACE: Global Registry of Acute Coronary Events; hs-CRP: high-sensitivity C-reactive protein; SYNTAX: Synergy Between PCI with Taxus and Cardiac Surgery; TC: total cholesterol; eGFR: estimated glomerular filtrating rate; HbA1c: hemoglobin A1c; SUA: serum uric acid; CKD: chronic kidney disease.

**Table 2.** Study quality evaluation *via* the Newcastle-Ottawa Scale.

Study	Representativeness of the exposed cohort	Selection of the non-exposed cohort	Ascertainment of exposure	Outcome not present at baseline	Control for age and sex	Control for other confounding factors	Assessment of outcome	Enough long follow-up duration	Adequacy of follow-up of cohorts	Total
Hartopo 2016	1	1	1	1	0	0	0	1	1	6
Ma 2020	1	1	1	1	1	1	1	1	1	9
Zheng 2022	1	1	1	1	1	1	1	1	1	9
Shao 2022	1	1	1	1	1	1	1	1	1	9
Liu 2023	0	1	1	1	1	1	1	1	1	8
Wang 2023	0	1	1	1	1	1	1	1	1	8
Ozen 2023	1	1	1	1	1	1	1	1	1	9
Kan 2023	1	1	1	1	1	1	1	1	1	9
Karre 2024	0	1	1	1	0	0	1	1	1	6

### Meta-analysis for the association between AIP and MACE in ACS patients

**Figure 2.**
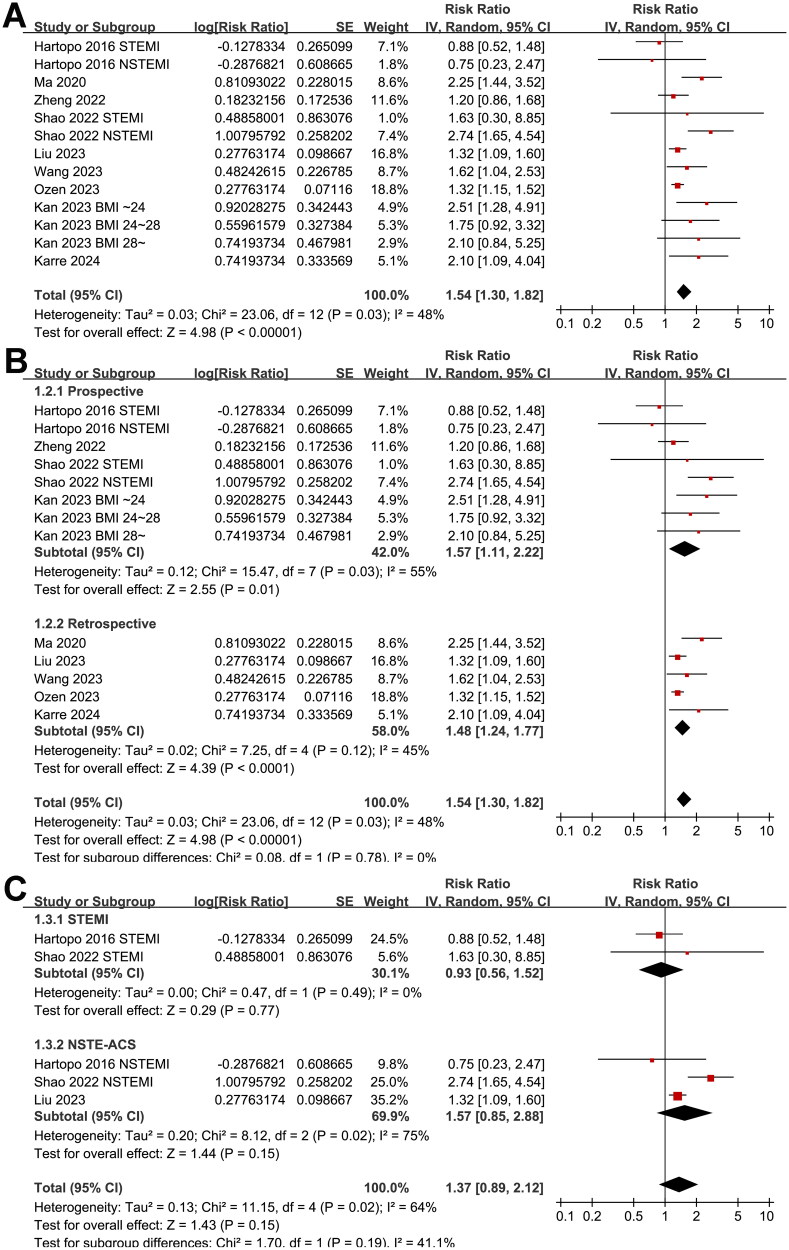
Forest plots for the meta-analysis of the association between AIP and the risk of MACE in patients with ACS; (A) forest plots for the overall meta-analysis; (B) forest plots for the subgroup analysis according to study design; and (C) forest plots for the subgroup analysis according to the subtype of ACS.

Since two studies reported the results in patients with STEMI and NSTEMI separately [11,13], and another study reported the outcome according to the body mass index of the patients [15], these datasets were included in the meta-analysis independently. Finally, thirteen datasets from nine cohort studies [11–19], involving 10861 patients with ACS were included in the meta-analysis. Pooled results with a random-effects model showed that, compared to the ACS patients with the lowest category of AIP at admission, those with the highest category of AIP had a higher incidence of MACE during follow-up (RR: 1.54, 95% CI: 1.30–1.82, *p* < 0.001; Figure 2A) with moderate statistical heterogeneity (*I*^2^ = 48%). Sensitivity analysis by excluding one dataset at a time retrieved consistent results (RR: 1.44–1.61, *p* all <0.05).

**Figure 3.**
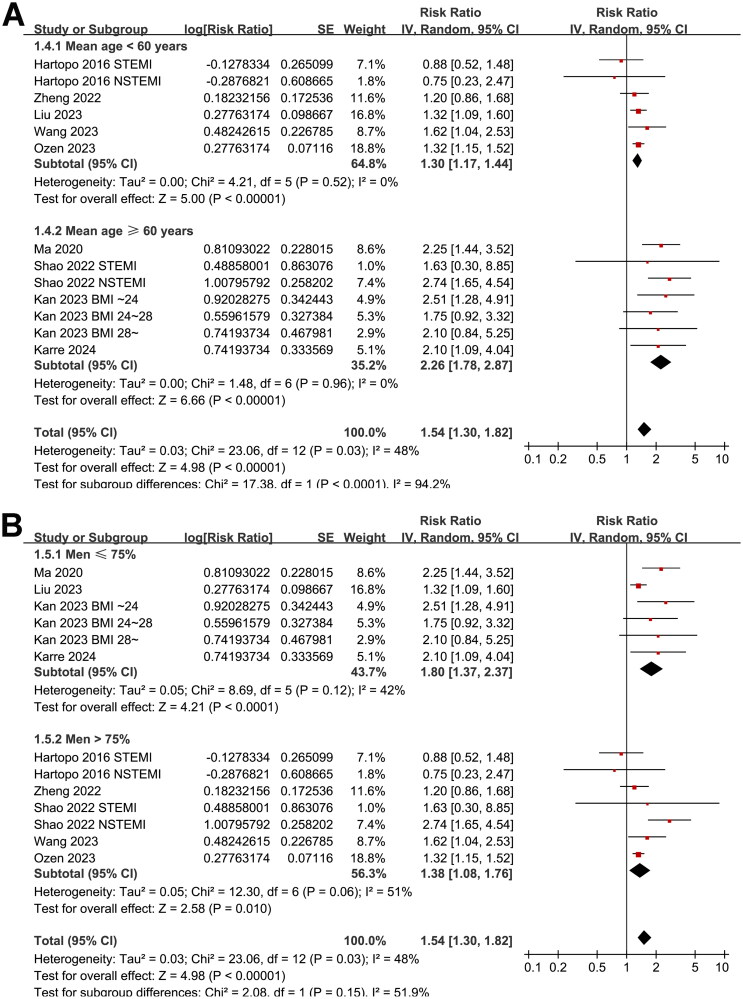
Forest plots for the subgroup analyses of the association between AIP and the risk of MACE in patients with ACS; (A) forest plots for the subgroup analysis according to age of the patients; and (B) forest plots for the subgroup analysis according to the proportion of men.

**Figure 4.**
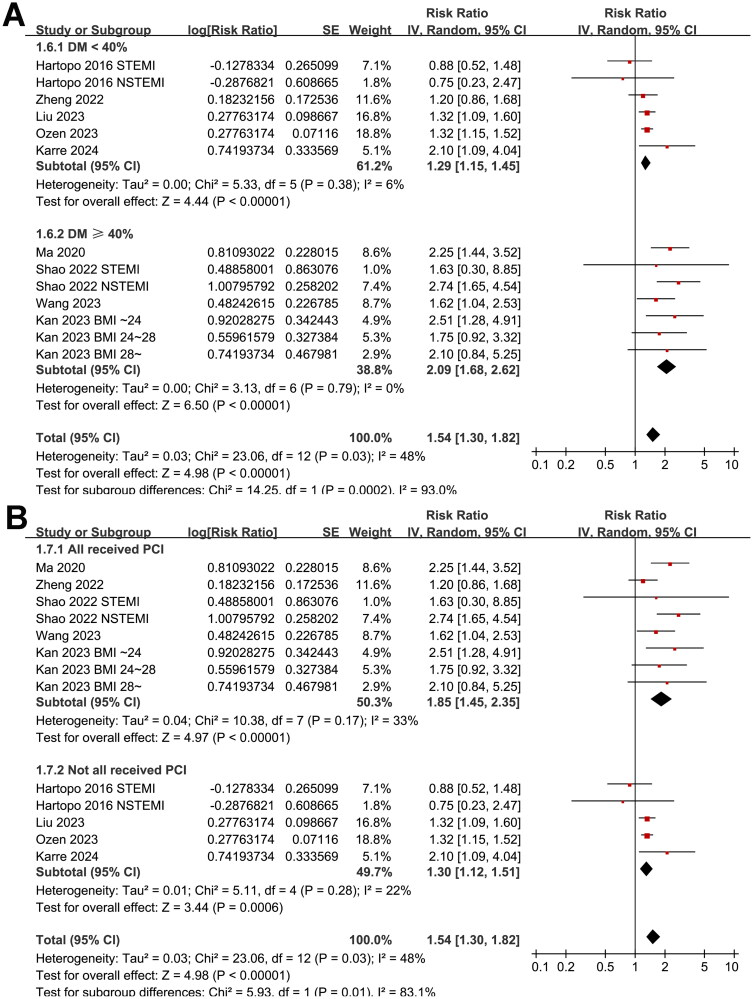
Forest plots for the subgroup analyses of the association between AIP and the risk of MACE in patients with ACS; (A) forest plots for the subgroup analysis according to the proportion of patients with diabetes; and (B) forest plots for the subgroup analysis according to the proportion of patients who were treated with PCI.

**Figure 5.**
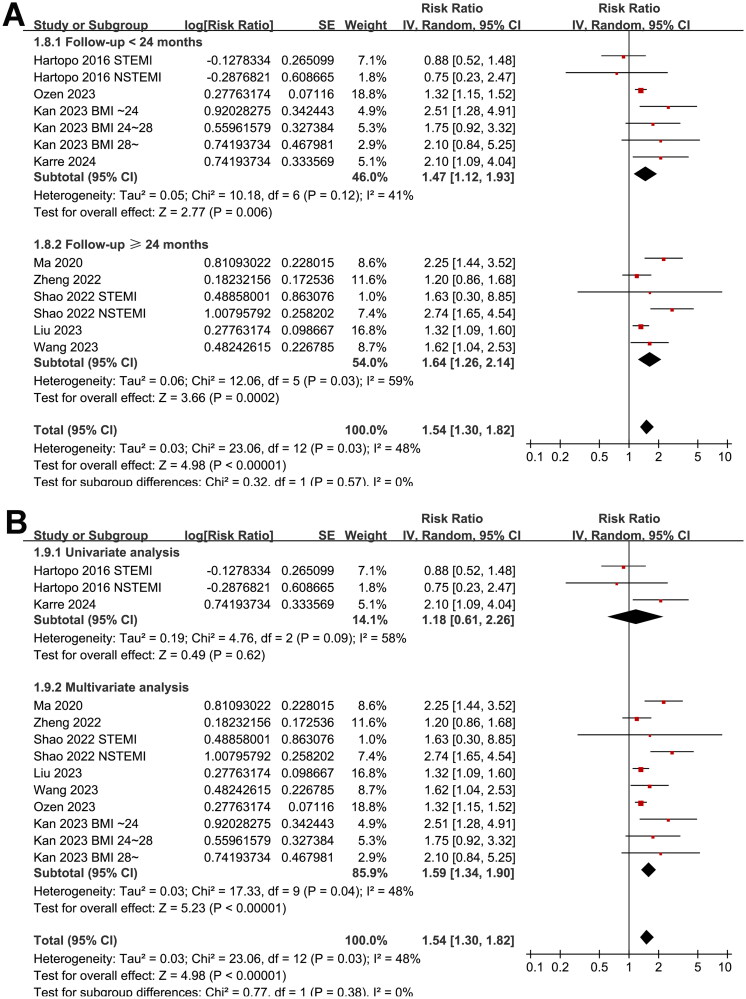
Forest plots for the subgroup analyses of the association between AIP and the risk of MACE in patients with ACS; (A) forest plots for the subgroup analysis according to the follow-up duration; and (B) forest plots for the subgroup analysis according to the analytical model (univariate or multivariate).

Further subgroup analyses suggested that the association between AIP and MACE was not significantly affected by study design (*p* for subgroup difference = 0.78, Figure 2B), and the results were not significantly different in patients of STEMI and NSTEACS (*p* for subgroup difference = 0.19, Figure 2C). Interestingly, the subgroup analysis suggested a stronger association between a high AIP and an increased risk of MACE in older patients (mean age ≥60 years, RR: 2.26, 95% CI: 1.78–2.87, *p* < 0.001; *I*^2^ = 0%) than the younger ones (mean age <60 years, RR: 1.30, 95% CI: 1.17–1.44, *p* < 0.001; *I*^2^ = 0%; *p* for subgroup difference <0.001; Figure 3A), which fully explained the heterogeneity. The subgroup analysis according to the proportion of men showed consistent results (*p* for subgroup difference = 0.15, Figure 3B). Further subgroup analyses showed a stronger association in studies with more patients with diabetes (≥40%, *p* for subgroup difference <0.001; Figure 4A), and in studies of patients who were all treated with PCI (*p* for subgroup difference = 0.01; Figure 4B). Finally, the results of subgroup analysis did not show that the follow-up duration (*p* for subgroup analysis = 0.57; Figure 5A) and analytic model (*p* for subgroup analysis = 0.38; Figure 5B) may significantly modify the association between AIP and MACE.

### Publication bias evaluation

**Figure 6.**
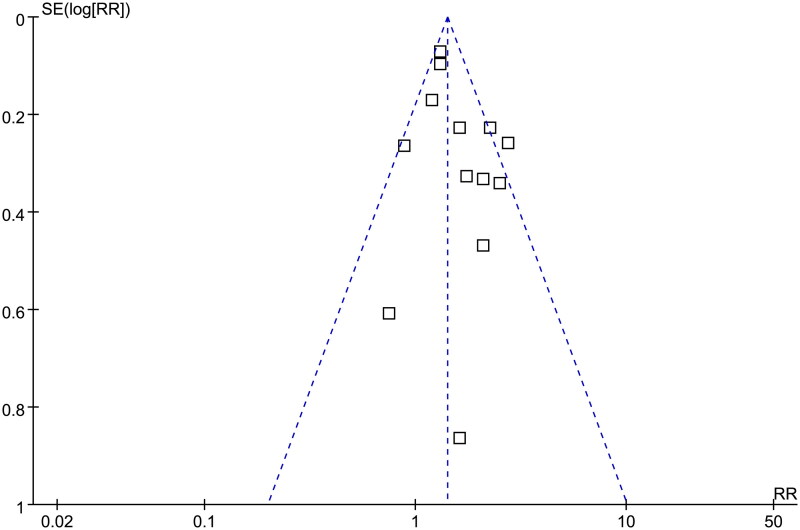
Funnel plots for the publication bias underlying the meta-analysis of the association between AIP and the risk of MACE in patients with ACS.

The funnel plots for the meta-analysis of the association between AIP and MACE in ACS patients are shown in Figure 6. The symmetrical nature of the funnel plots suggested a low likelihood of publication bias. The result of Egger’s regression test also showed a low risk of publication bias (*p* = 0.64).

## Discussion

This meta-analysis examined 13 datasets derived from nine cohort studies and found that patients with ACS and a high AIP upon admission had a notably higher risk of MACE during follow-up compared to those with a low AIP. The subsequent sensitivity analysis consistently upheld these results. Additionally, subgroup analysis indicated that older patients (mean age ≥60 years) showed a stronger correlation between high AIP and increased MACE risk than did younger patients (mean age <60 years), thus explaining the source of heterogeneity in the data. The findings also suggested a stronger association between AIP and MACE in studies where more patients had diabetes (≥40%) and in studies where all patients underwent PCI treatment. In summary, this meta-analysis implies that there is an association between high AIP levels and an elevated risk of MACE in ACS patients, particularly among older individuals.

During the preparation of the manuscript, a meta-analysis was published which showed that a high AIP was associated with poor clinical outcomes in patients with CAD [26]. Both the patients with stable CAD and ACS were included in this study; while a subgroup analysis was not performed, leaving uncertainty regarding the association between AIP and MACE in patients with ACS [26]. Moreover, both cross-sectional and cohort studies were included in the previous meta-analysis, making the interpretation of the results difficult [26]. Our study has several methodological strengths compared to the previous meta-analysis. We conducted a comprehensive search of three commonly used electronic databases focusing on the role of AIP in patients with ACS, leading to the identification of nine relevant cohort studies for inclusion in this meta-analysis. All studies included were longitudinal cohort studies capable of establishing a link between high AIP and an increased likelihood of MACE in these patients. Additionally, we conducted multiple sensitivity and subgroup analyses to validate the findings and explore potential sources of variation among the studies. Importantly, consistent results were obtained in sensitivity analysis when each dataset was removed individually, suggesting that no single dataset disproportionately influenced the outcomes. Additionally, subgroup analysis indicated a stronger correlation between a high AIP and an elevated risk of MACE in older patients compared to younger ones. While the reasons for this finding are not fully understood, a further subgroup analysis revealed that the association is also more pronounced in studies with a higher proportion of patients with diabetes. These results suggest that AIP may serve as a significant indicator of increased MACE risk in patients with ACS having higher risk profiles, including both older individuals and those with diabetes. Furthermore, consistent findings were observed in studies employing multivariate analyses, implying that the link between high AIP and increased MACE risk in patients with ACS is likely independent of traditional cardiovascular risk factors. These conclusions support the use of AIP as a prognostic factor among ACS patients, particularly within high-risk subgroups, such as older individuals and those with diabetes.

There are likely several different mechanisms that may contribute to the link between a high AIP and the elevated risk of MACE in individuals with ACS. From a pathological perspective, smaller and denser particles are more prone to oxidation and have greater potential for causing atherosclerosis [27]. Previous studies have suggested that AIP is associated with lipoprotein particle size, density, and peroxidation rates, making it a dependable marker of plasma atherogenicity [9,28]. Moreover, increasing evidence indicates that high AIP levels are connected to the extent of coronary artery stenosis [29,30] and pre-PCI thrombolysis in myocardial infarction flow [31], indicating that AIP may reflect the severity of coronary lesions in ACS patients. Furthermore, an increased AIP has also been linked to a higher risk of slow coronary flow [32], no-reflow phenomenon [33], and in-stent restenosis [34]—factors which could lead to worse prognoses for those with ACS.

The potential mechanisms underlying the influence of AIP on MACE in patients with ACS rely on the role of TG and HDL-C. Currently, the potential molecular and pathophysiological mechanisms underlying the association between AIP and MACE after ACS remain poorly understood. A few hypotheses may be helpful for future exploration. First, a high AIP reflects an imbalance in lipid metabolism, characterized by elevated levels of TG and/or decreased levels of HDL-C, which promotes the formation of atherosclerotic plaques [35]. Second, dyslipidemia associated with a high AIP contributes to systemic inflammation, which plays a crucial role in the progression of atherosclerosis and destabilization of coronary plaques, leading to ACS [36]. Third, high AIP levels may impair endothelial function, leading to reduced nitric oxide bioavailability, increased oxidative stress, and enhanced endothelial adhesion molecule expression, all of which promote plaque formation and thrombosis [37]. Finally, dyslipidemia associated with high AIP levels can promote a pro-thrombotic state through various mechanisms, including platelet activation [38], increasing the risk of coronary thrombosis, and MACE. Additional studies are warranted to determine the key molecular signaling pathways underlying the association between a high AIP and an increased risk of MACE in patients with ACS.

The study has some limitations. Five of the included studies were retrospective, which may introduce biases in selection and recall that may have influenced the results. However, subgroup analysis in the prospective studies showed consistent findings. As an important component of AIP, serum triglyceride level is closely related to the fasting duration. Although all of the included studies stated the measurement of TG at a fasting state, information on fasting duration is generally not reported. In addition, none of the included studies reported the TG values before admission to reflect the long-term TG values. Further studies should incorporate the potential influence of fasting duration on the association between AIP and MACE. Additionally, there was variability in the cutoff values for AIP among the included studies, leading to heterogeneity. Further research is necessary to establish an optimal cutoff for AIP to predict MACE risk in these patients. While subgroup analysis restricted to studies with multivariate analyses yielded similar results, unadjusted confounding factors may still have impacted the association. Finally, our study relied solely on observational research; therefore, a definitive causal link between high AIP and increased incidence of MACE in patients with ACS could not be conclusively established. It will be interesting for future studies to investigate if reducing AIP could further improve the prognosis of patients with ACS, especially for the patients who have achieved the treatment target for their LDL-C.

## Conclusions

The results of this meta-analysis suggest that ACS patients with a high AIP upon admission may experience a greater incidence of MACE during their follow-up when compared to those with low AIP. While further validation in large-scale prospective studies is necessary and the underlying mechanisms require exploration, these findings endorse the potential utilization of AIP as a prognostic indicator for ACS patients.

## Supplementary Material

PRISMA_2020_checklist (4).docx

## Data Availability

The authors confirm that the data supporting the findings of this study are available within the article.
